# Social Service in America

**Published:** 1921-03-19

**Authors:** N. F. Cummings

**Affiliations:** Managing Editor, *Hospital Social Service*, 19 E. 72nd Street, New York


*To the Editor of* The Hospital.

### Social Service in America.

*To the Editor of* The Hospital.

Sir,—I was very interested in the letter from "A Social Worker" in the issue of January 29. I think it is very much to the point, as is her fresh and original picture of the birds of passage who light in the homes of our case studies and then flit on to their central desk that the last items may be added to the records.

We do have a multiplicity of organisations and of specialised workers. I believe that the pendulum will swing far in the other direction in the future. One of our leading medical men said in a discussion of institutional classes lately: "Let us cease to stigmatise the children in the out-patient departments with pathological terms and enrol them under health classes, for health is the real objective, as well as cardiac, tuberculosis, and nutritional work."

We wish very much to bring into our pages some of the points of view of your group of workers, as we need the interchange of experience, for the lady almoners of the English hospitals were the original hospital social workers.—Yours faithfully,

N. F. Cummings,

Managing Editor, *Hospital Social Service*,

19 E. 72nd Street, New York.

